# Small Molecule Binding to Alzheimer Risk Factor CD33 Promotes Aβ Phagocytosis

**DOI:** 10.1016/j.isci.2019.07.023

**Published:** 2019-07-19

**Authors:** Luke A. Miles, Stefan J. Hermans, Gabriela A.N. Crespi, Jonathan H. Gooi, Larissa Doughty, Tracy L. Nero, Jasmina Markulić, Andreas Ebneth, Berthold Wroblowski, Daniel Oehlrich, Andrés A. Trabanco, Marie-Laure Rives, Ines Royaux, Nancy C. Hancock, Michael W. Parker

**Affiliations:** 1 ACRF Rational Drug Discovery Centre, St. Vincent's Institute of Medical Research, Fitzroy, VIC 3056, Australia; 2 Department of Biochemistry and Molecular Biology, Bio21 Molecular Science and Biotechnology Institute, University of Melbourne, Parkville, VIC 3010, Australia; 3 Janssen Research & Development, a Division of Janssen Pharmaceutica N.V, 2340 Beerse, Belgium; 4 Neuroscience Medicinal Chemistry, Janssen Research & Development, 45007 Toledo, Spain; 5 Molecular and Cellular Pharmacology, Janssen Research & Development, LLC, La Jolla, CA 92121, USA

**Keywords:** Molecular Structure, Neuroscience, Molecular Neuroscience, Components of the Immune System, Protein Structure Aspects

## Abstract

Polymorphism in the microglial receptor CD33 gene has been linked to late-onset Alzheimer disease (AD), and reduced expression of the CD33 sialic acid-binding domain confers protection. Thus, CD33 inhibition might be an effective therapy against disease progression. Progress toward discovery of selective CD33 inhibitors has been hampered by the absence of an atomic resolution structure. We report here the crystal structures of CD33 alone and bound to a subtype-selective sialic acid mimetic called P22 and use them to identify key binding residues by site-directed mutagenesis and binding assays to reveal the molecular basis for its selectivity toward sialylated glycoproteins and glycolipids. We show that P22, when presented on microparticles, increases uptake of the toxic AD peptide, amyloid-β (Aβ), into microglial cells. Thus, the sialic acid-binding site on CD33 is a promising pharmacophore for developing therapeutics that promote clearance of the Aβ peptide that is thought to cause AD.

## Highlights

• A sialic acid mimetic increases the uptake of Alzheimer peptide into microglia
• Crystal structure of cell surface receptor CD33 bound to the sialic acid mimetic
• Crystal structure of unliganded CD33 and mutagenesis studies revealed key residues
• CD33 carbohydrate-binding site is a pharmacophore for Alzheimer drug development

## Graphical Abstract

**Figure:**
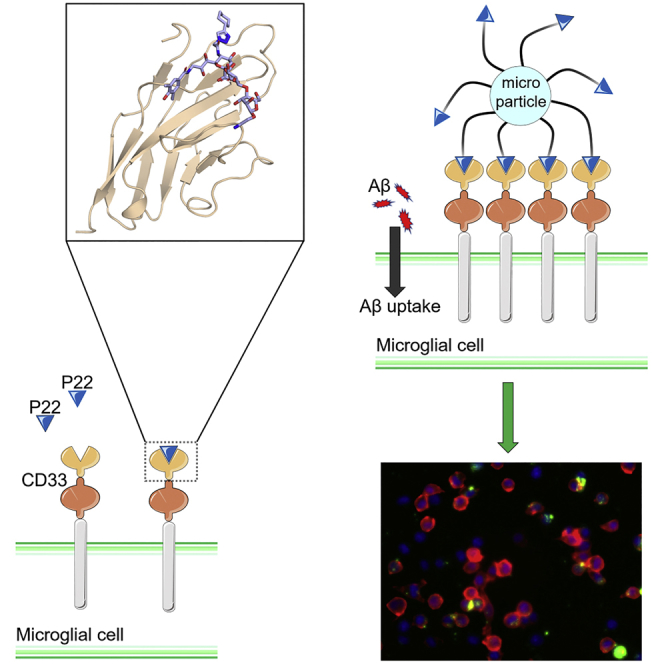

## Introduction

Microglia, resident immune cells in the brain, can be activated in response to misfolded proteins found in neurodegenerative diseases leading to neuroinflammation and the release of neurotoxic substances. There is emerging evidence that microglial activation may play an important role in a range of neurodegenerative diseases including Huntington disease, Parkinson disease, amyotrophic lateral sclerosis, frontotemporal dementia, and dementia with Lewy bodies (Heneka et al., 2014, Leyns and Holtzman, 2017, Hickman et al., 2018). In a cell-based model of Alzheimer disease (AD) it has been shown that activated microglia can induce neurite degeneration and cell death, a pathological feature of the disease (Park et al., 2018).

Many of the recently identified genes associated with late-onset AD risk are integral to the innate immune system (Sims et al., 2017, Efthymiou and Goate, 2017). Some of these genes code for microglial proteins, such as the strongest genetic risk factor for AD, namely APOE, and the cell surface receptor CD33 (Krasemann et al., 2017, Pimenova et al., 2017). Genome-wide association studies of late-onset AD demonstrate a link between the CD33 gene and disease susceptibility (Hollingworth et al., 2011, Naj et al., 2011). Tanzi and co-workers showed that knocking out the CD33 gene could mitigate amyloid-β (Aβ42) pathology (Griciuc et al., 2013). They also reported that numbers of CD33-immunoreactive microglia correlated positively with both insoluble Aβ42 levels and amyloid plaque burden in AD brain, that CD33 inhibited clearance of Aβ42 in microglial cell cultures, and that brain levels of insoluble Aβ42/plaque burden were markedly reduced in APPSwe/PS1ΔE9/CD33−/− mice. They also showed that the SNP rs386544, which confers protection from AD, lowered insoluble levels of Aβ42 in the AD brain. The rs386544 allele is in perfect linkage disequilibrium with rs12459419, which is located at a splice site of the sialic acid-binding site containing exon 2 (Raj et al., 2014). Both alleles ultimately lead to lower expression levels of functional CD33 (Malik et al., 2013, Walker et al., 2015), enhanced phagocytic activity of microglial cells, and uptake of Aβ42 (Griciuc et al., 2013, Bradshaw et al., 2013). The widely accepted “amyloid” hypothesis for AD posits that increased production and oligomerization of the Aβ42 peptide initiates a cascade of events leading to neurodegeneration and AD. Thus, small, drug-like inhibitors of CD33 to promote amyloid clearance could represent a novel class of therapeutics for the prevention and treatment of AD.

CD33 is a pattern recognition receptor belonging to the Siglec (sialic acid-binding Ig-like lectins) receptor family. It is a type-1 membrane protein with an extracellular region consisting of an N-terminal V-set domain that recognizes sialylated ligands and an Ig juxtamembrane (C2-set) domain (Griciuc et al., 2013, Rillahan et al., 2014). Although CD33 recognizes sialylated glycoproteins and gangliosides that extensively coat amyloid plaques, its natural ligand has not been identified and up until recently known ligands had only millimolar affinity. Paulson and co-workers recently identified sialic acid-based ligands with high selectivity and micromolar affinity for human CD33 (Rillahan et al., 2014). To provide a molecular basis for understanding ligand specificity and facilitate the design of drug-like inhibitors of CD33 activity, we have solved high-resolution (1.8-Å) crystal structures of the human CD33 V-set domain, with and without a CD33-selective 2,5,9-trisubstituted sialic acid mimetic called P22 (Figure S1). Importantly, we show that P22 binding to the sialic acid-binding pocket of CD33 can increase uptake of the toxic Aβ42 peptide into microglial cells, and hence CD33 is a promising target for structure-based drug discovery for the treatment of AD.

## Results and Discussion

### Crystal Structure of the P22-CD33 Complex

We collected separate X-ray diffraction data from two P22-CD33 complex crystals, which we refer to as Crystal no. 1 (corresponding to P22 binding mode 1) and Crystal no. 2 (corresponding to P22 binding mode 2) in Table S1 and solved their structures. In each crystal, two complexes were found in the asymmetric unit, designated by their chain identifiers A and B, and they overlay very closely with root-mean-square deviation (RMSD) over Cα atoms of 0.27 and 0.29 Å for the two structures (Figure S2).

**Figure 1:**
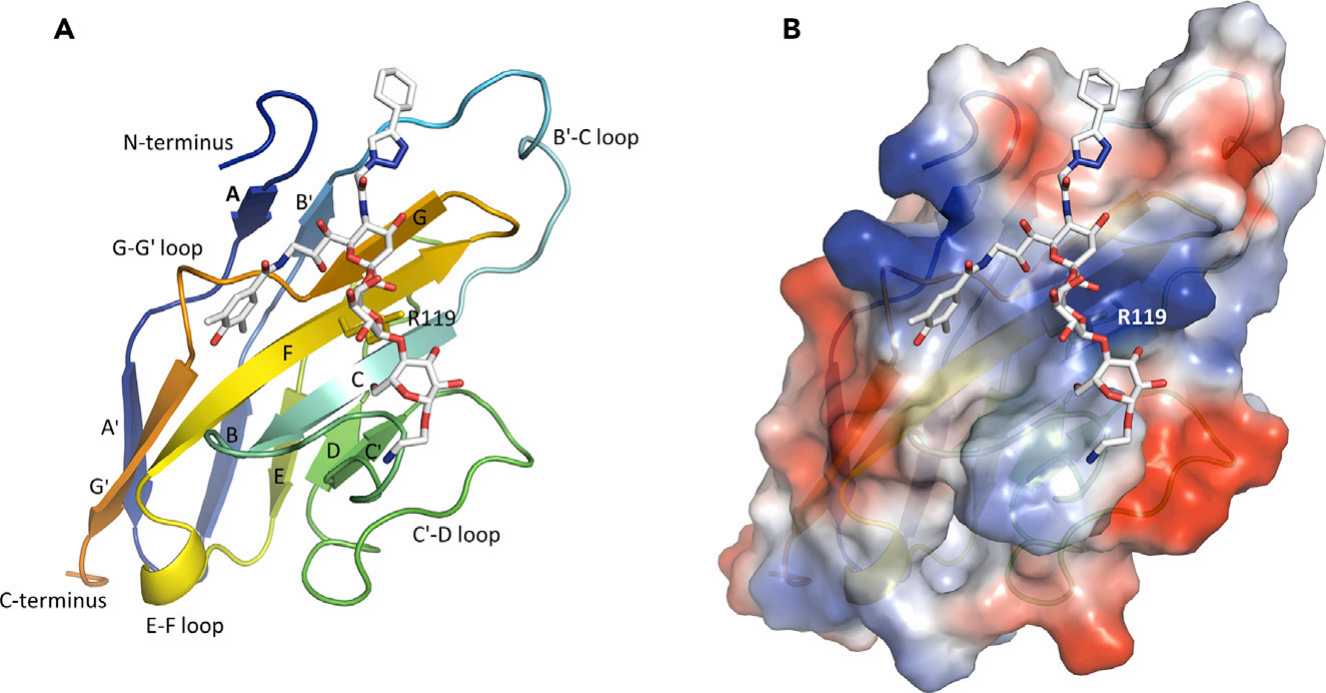
Structure of the P22-CD33 Complex (A) Cartoon representation of the CD33 N-terminal V-set domain in rainbow coloring from blue (N terminus) to orange (C terminus). The critical sialic acid-binding residue R119 (colored orange) and compound P22 (gray) are shown as sticks. (B) The electrostatic potential energy mapped onto the molecular surface of CD33 is shown, in the same orientation as (A), with electropositive regions colored blue and electronegative regions red. The location of R119 is indicated. See also Figure S3 and Table S1.

The crystal structure of P22 bound to the CD33 V-set domain is shown in Figure 1 (see Transparent Methods). The domain forms a typical Siglec V-set domain fold, rich in β-sheet and composed of 11 β-strands (A, A′, B, B′, C, C′, D, E, F, G, and G′). There is an intra-domain disulfide bond between C41 and C101. Comparison with the V-set domains of other human Siglec subtypes available in the Protein DataBank (Figure S3) reveals very similar folds with the B′-C, C-C′, C′-D, and G-G′ loops being the most distinguishing features, exhibiting little or no sequence similarity with the other human Siglecs.

P22 exhibits high specificity for human CD33 over other human Siglecs (Rillahan et al., 2014). We observe two P22 binding modes, with one showing more interactions and straddling the length of one face of CD33 (Figure 1) burying a surface area of 509 Å^2^. Although the space group was the same and the cell dimensions were very similar between the two crystal forms, the location of the C2-substituent in the CD33 carbohydrate-binding pocket is different. This substituent is highly mobile and shows little or no engagement with CD33 directly (see Figure S4). In binding mode 1 the surface buried by P22 is 509 Å^2^, with the C2-substituent within hydrogen bonding distance of the backbone carbonyl and side-chain hydroxyl of S68. The B-factor of the C2-substituent is over 55 Å^2^, whereas the B-factors of the C5- and C9-substituents, and contacting side-chains, are less than 35 Å^2^. In binding mode 2 the buried surface area is 370 Å^2^, with the C2-substituent making no contact with the protein, but it does form interactions with another CD33 molecule in the crystal lattice. The B-factors of the C2-substituent are over 45 Å^2^, whereas the B-factors of the C5- and C9-substituents are less than 30 Å^2^ and those of the contacting side-chains are less than 25 Å^2^. We chose to perform all our subsequent structural analyses on the binding mode 1 structure (Crystal no. 1).

The P22 molecules themselves within each crystal form superimpose very closely (RMSD over all heavy atoms of 0.32 Å in Crystal form no. 1 and 0.46 Å in Crystal form no. 2). Despite the overall similarity of the two complexes there are a few subtle differences. Several amino acid side-chains are surface exposed and exhibit flexibility between molecules, such as K52, R98, H137, and K130. Several residues are packed against a molecule from an adjacent asymmetric unit resulting in side-chain movement to accommodate the close packing; these are W22, L78, Q81, and R91. In the case of R91, side-chain movement facilitates the formation of additional hydrogen bonds to E27 of another asymmetric unit. Aside from these minor differences, the four molecules of CD33 bound to P22 across our two reported structures are essentially identical.

**Figure 2:**
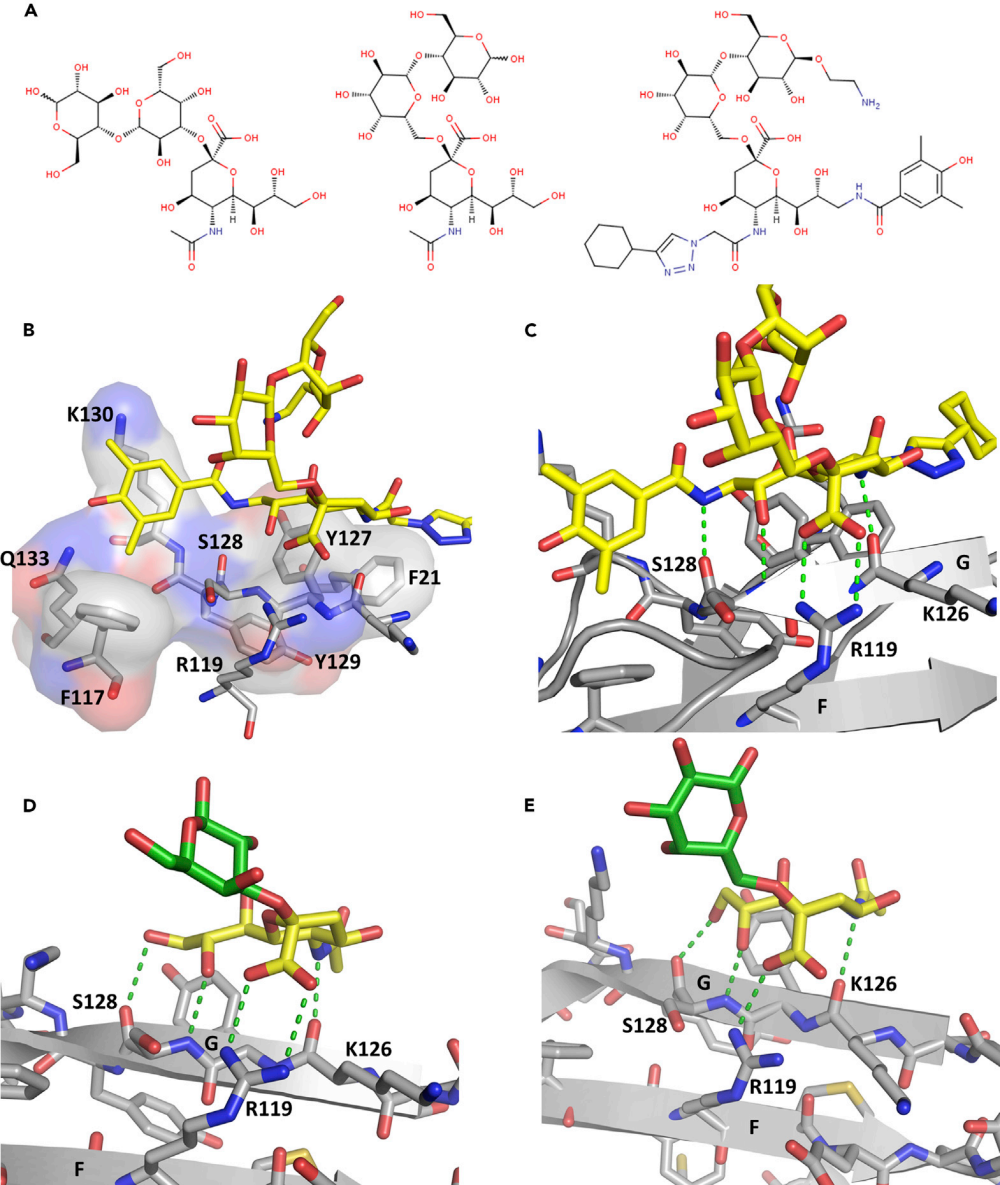
P22 Interactions with CD33 (A) The chemical structures of 3′-sialyllactose, 6′-sialyllactose, and P22 from left to right, respectively. The sialic acid moiety of the three ligands is shown in the same view. (B) Close-up view of the CD33 residues (gray sticks) directly interacting with P22 (yellow sticks). Residues making hydrophobic interactions with P22 are overlayed with a semi-transparent molecular surface. (C) The extended polar contact network (green dashes) P22 makes with the F- and G-strands of CD33. (D and E) (D) 3′-sialyllactose and (E) 6′-sialyllactose binding to the key CD33 R119 residue. The sialic acid moiety of the ligand is shown in yellow and the galactopyranosyl ring in green. Polar contacts between the ligand and CD33 are indicated by the green dashes. In both the 3′- and 6′-sialyllactose complexes with CD33, the glucopyranosyl ring is not visible (PDB: 5J06 and 5J0B). See also Figures S1, S2, and S4 and Table S2.

Interactions between P22 and CD33 observed across all structures reported here include one salt bridge, three hydrogens bonds, and numerous van der Waals interactions with four surrounding residues (Table S2). The central sialic acid moiety is engaged by R119 in a salt bridge (Figure 2C). R119 is a highly conserved residue in Siglecs, located in a highly basic pocket in CD33 (Figures 1B and S3D). Other P22 polar interactions include hydrogen bonds contributed by the main-chain atoms of K126 and S128. A notable van der Waals packing interaction occurs between the C5- and C9-substituents of P22 and an aromatic knob consisting of F21, H45, and Y127 (Figure 2B). The C9-substituent of P22 extends away from the sialic acid-binding site along the G-strand, past the G-G′ loop (Figure 1A). Interestingly, the sequence in this region (TKYSYKSPQ) shows no similarity with other human Siglecs (Figure S3D), suggesting a molecular basis for the affinity and selectivity of CD33 for P22 (Rillahan et al., 2014).

### P22 Binds CD33 with Micromolar Affinity

**Figure 3:**
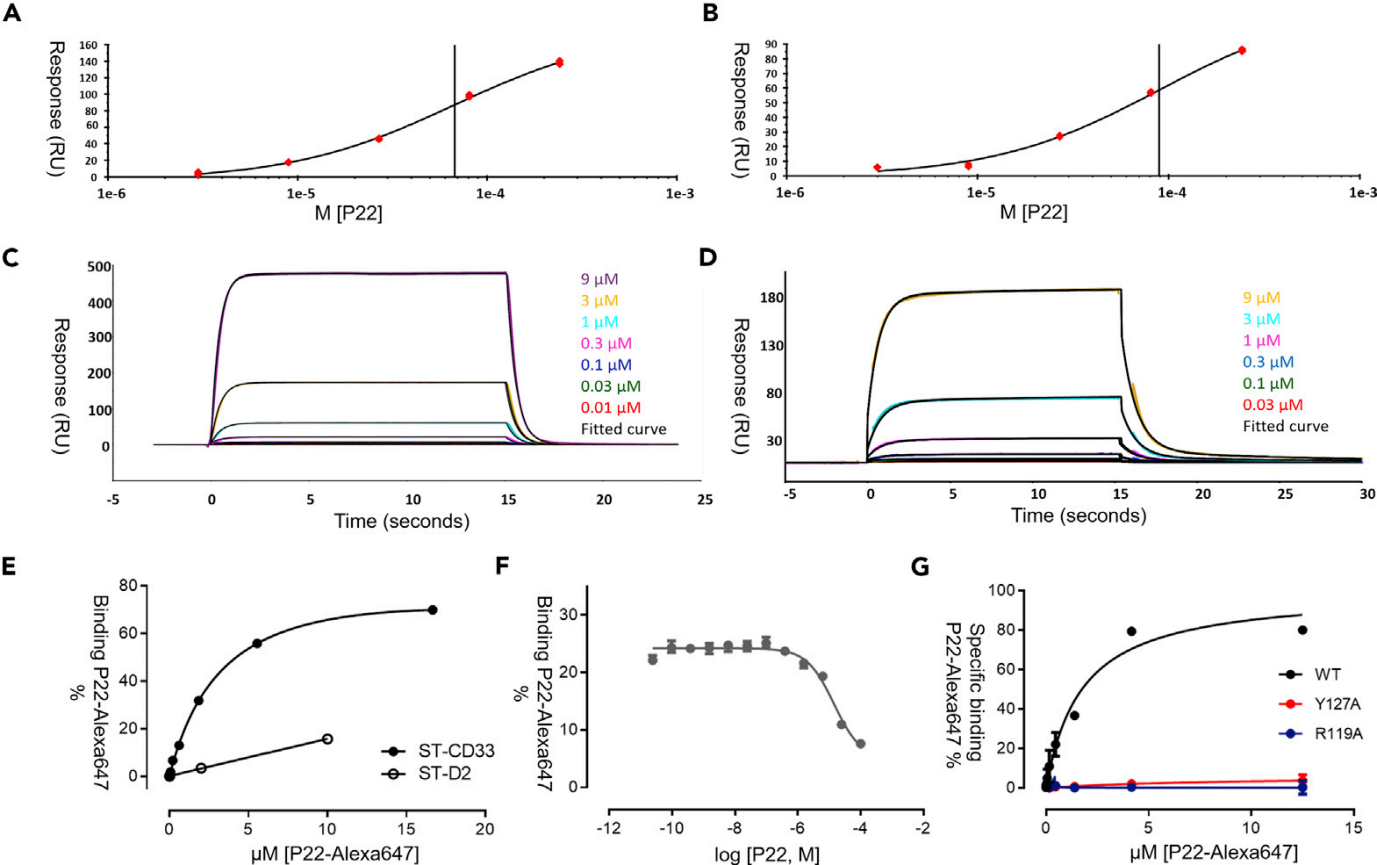
P22 Binding to CD33 Wild-Type and Mutants (A) An example of affinity analysis for 1–243 μM (3-fold dilutions) of P22 binding to immobilized CD33^C36S^. Data obtained in duplicate using single-cycle analysis and fit to a steady-state binding model to determine the equilibrium binding constant. (B) An example of affinity analysis of P22 binding to immobilized CD33 wild-type (WT) as per example in (A). (C) Sensorgram data for 0.01–9 μM (3-fold dilutions) of CD33^C36S^ binding to immobilized P22. Data obtained in triplicate using single concentration cycles and fit to a 1:1 binding model for kinetic analysis. (D) Sensorgram data for 0.03–9 μM (3-fold dilutions) CD33 WT binding to immobilized P22. Data obtained in triplicate using single concentration cycles and fit to a bivalent analyte model for kinetic analysis. Measurements in triplicate showing mean ± SD. (E) Saturation total binding experiments on either CD33 (black circles) or the dopamine D2 receptor (open circle) showing that P22-Alexa647 binds specifically to CD33. Data presented are representative of three independent experiments performed in quadruplicate for each compound. Data were normalized on P22-Alexa647 estimated B_max_ on CD33 and are represented as averages ± SD. (F) Competition binding experiment on CD33 using 2 μM P22-Alexa647. Data presented are representative of three independent experiments performed in quadruplicate for each compound. Data were normalized on P22-Alexa647 estimated B_max_ on CD33 and are represented as averages ± SD. (G) The specific binding of P22-Alexa647 (total binding – binding in presence of 100 μM non-labeled P22) was evaluated for the WT CD33 and two other single point mutation variants. Binding was completely abolished by the Y127A and R119A variants. Data presented are representative of three independent experiments performed in quadruplicate for each compound. Data were normalized on P22-Alexa647 estimated B_max_ on CD33 and are represented as averages ± SD. See also Tables S3 and S4.

Paulson and co-workers estimated an IC_50_ for P22 of 11 μM using a flow cytometry assay (Rillahan et al., 2014). We next performed surface plasmon resonance (SPR) measurements on P22 binding to CD33 wild-type, and selected mutants, to obtain detailed kinetic data (see Transparent Methods). The CD33 V-set domain has a free cysteine residue (C36), which caused anomalous results in some of our SPR experiments due to disulfide-linked oligomers forming in solution. Hence, we mutated this cysteine to serine for these experiments. The *K*_*D*_ for P22 binding to captured CD33^C36S^ was determined by SPR as 95 ± 39 μM (close to the wild-type value of 118 ± 41 μM) (Figure 3 and Table S3). With biotinylated P22 (Figure 3C) captured on the chip, analysis of CD33^C36S^ binding to P22 yielded a *K*_*D*_ of 67 ± 15 μM (Table S3). In either orientation, no binding was observed for CD33 when the key R119 was mutated to alanine.

### Crystal Structure of Unliganded CD33

We determined the crystal structure of the unliganded CD33 V-set domain to answer the question whether CD33 recognizes carbohydrate ligands using an induced fit mechanism. Our crystals of unliganded CD33 contain four copies of the molecule in the asymmetric unit, chains A to D. They are assembled as non-biological dimers, AB and CD, with a free cysteine, C36, forming a disulfide bond between monomers. This cysteine would normally form an inter-domain disulfide with C169 of the C2 domain in the intact receptor. Each CD33 molecule superimposed closely with each other (maximum RMSD of 0.39 Å over 115 Cα atoms) with the largest deviations resulting from side-chain movements to accommodate the packing of the two non-biological dimers in each asymmetric unit. Residues W22, Q24, and F44 show equivalent side-chain positions in chains A and B, with an alternative position in chains C and D, to facilitate packing of the asymmetric unit. Additional differences between each CD33 molecule of the asymmetric unit are observed in surface-exposed and flexible amino acids, such as E27, K52, R69, L78, R98, R111, and K130. As the unliganded structures are so similar all further comparisons in the text focus on chain A as the reference unliganded CD33 molecule. Each of the four unliganded structures superimpose closely on the P22 crystal structures (maximum RMSD of 0.64 Å over 116 Cα atoms) (Figure S2C).

The unliganded CD33 superimposes closely on the P22 complex crystal structure. The most significant difference between the structures in the P22-binding pocket is in the aromatic cluster consisting of F21, H45, and Y127 (Figure S2). On binding P22, the H45 side chain rotates down about the Cβ carbon to accommodate F21 in the binding site with the latter forming an aromatic π-stack with Y127.

The crystal structures of the entire extracellular region of CD33 bound to two sialic acid derivatives (3′- and 6′-sialyllactoses see Figure 2A) have been deposited in the Protein DataBank (PDB: 5J06, 5J0B) but have not yet been published. The V-set domain of the deposited structures and the ones here superimpose closely (maximum RMSD of 0.41 Å over 112 Cα atoms) (Figure S2). Superposition of the sialic acid-binding sites reveals that CD33 recognizes the sialic acid moieties in very similar ways, with R119 engaging the sialic acid by a salt bridge and main-chain atoms of K126 and S128 forming hydrogen bonds with the carbohydrate in every case. However, the sialyllactoses are much smaller than P22 (Figure 2A) and as a consequence do not form as many interactions with CD33 (Figure 2), consistent with the fact they bind CD33 with only millimolar affinity (Blixt et al., 2003).

### Structural Comparison of Siglec Structures

There are 14 other known human Siglecs, ranging in pairwise sequence identity with the CD33 V-set domain from 22% to 60% (Figure S3). The V-set domains from the published crystal structures all adopt the same fold but show some variation on superposition with CD33: RMSD on Cα atoms of 1.75 Å over 61 residues for Siglec-2 (PDB: 5VKJ) (Ereño-Orbea et al., 2017), 0.85 Å over 113 residues for Siglec-5 (PDB:2ZG2) (Zhuravleva et al., 2008), 0.38 Å over 93 residues for Siglec-7 (PDB: 1O7S) (Dimasi et al., 2004), and 0.73 Å over 106 residues for Siglec-8 (PDB: 2N7A) (Pröpster et al., 2016) (see Figure S3A).

We noted above that there is a movement of aromatic side-chains in the carbohydrate-binding pocket surrounding the C5-substituent when P22 binds to CD33 (and see Figure S2). This side-chain rearrangement is seen in some, but not all, CD33 molecules in the 3′- and 6′-sialyllactose complex structures (PDB: 5J06, 5J0B). Furthermore, it is not observed in unliganded and ligand-bound structures of human Siglec-2 (PDB: 5VKJ, 5VKM) (Ereño-Orbea et al., 2017), Siglec-5 (PDB: 2ZG1, 2ZG2, 2ZG3) (Zhuravleva et al., 2008), Siglec-7 (PDB: 1NKO, 2DF3, 2GFR, 2HRL) (Dimasi et al., 2004, Attrill et al., 2006a, Attrill et al., 2006b), or Siglec-8 (PDB: 2N7A, 2N7B) (Pröpster et al., 2016). However, all the ligands in these cases have very short C5-substituents compared with P22. In contrast, side-chain rearrangements are observed in the human Siglec-2, CD33, and Siglec-7 structures, where the C2- (Siglec-2 and 7) and C9- (CD33 and Siglec-7) substituents are reasonably long (Ereño-Orbea et al., 2017, Attrill et al., 2006a, Attrill et al., 2006b). Thus, we predict that the side-chain rearrangements we observe in CD33 may only occur with larger carbohydrate and mimetic ligands.

### Site-Directed Mutagenesis Reveals Key CD33-Binding Residues

We then used our structural data to design mutations of key residues interacting with P22 to assess which interactions were the most important for binding affinity. The residues were mutated into alanine, and the effect of these mutations on P22 binding affinity was assessed using a SNAP-tag homogeneous time-resolved fluorescence binding assay (see Transparent Methods and Figures 3E–3G). The R119A mutation, which destroys the salt bridge between P22 and CD33, was one of the most detrimental to binding (>30-fold loss) (Figure 2C and Table S4). Y127A also completely abolished P22 binding, which can be explained by its location between the C5- and C9-substituents of P22 (Figure 2B and Table S4). An F21A mutation saw a 6-fold loss in binding affinity, possibly due to a loss of edge-to-face π-π engagement of P22, due to the orientation of Y127 (the tyrosine residue might become more dynamic in the absence of the ring stack), or due to reduced protein stability as the A-strand becomes more flexible. H45 and S131 showed little to no significant impact on P22 binding affinity, suggesting that these residues weakly interact with P22 (Table S4).

### P22 Promotes Phagocytosis in a CD33-Dependent Manner

**Figure 4:**
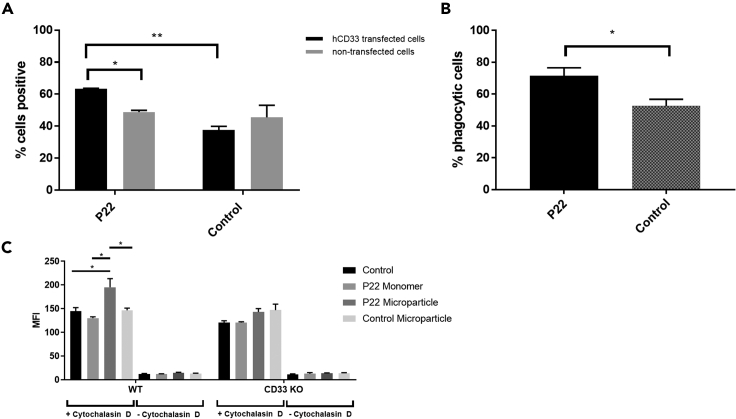
Effect of P22 on Phagocytosis The cells were treated with P22 conjugated to microparticles labeled with both streptavidin and fluorescein isothiocyanate (FITC), or with microparticles without P22 conjugated as control. (A) The percentage of positive cells for FITC microparticles was measured by flow cytometry, demonstrating that P22 preferentially binds to human CD33-transfected cells. Data were obtained in triplicate from n = 3 independent experiments and represented as mean ± SEM. *p < 0.05, **p < 0.01; ANOVA followed by the Tukey test. (B) Quantification of phagocytic human CD33-transfected cells for Aβ42 pHrodo after being incubated with microparticles reveals an increase of Aβ uptake for cells treated with P22. Data were obtained in triplicate from n = 3 independent experiments and represented as mean ± SEM. *p < 0.05; t test. (C) Quantification of phagocytic differentiated human THP-1 cells after incubation with P22 monomer, P22 conjugated microparticles, or microparticles without P22 conjugation. Differentiated THP-1 cells were either left untreated or were pre-treated with P22 monomers, P22 conjugated to microparticles, or microparticles alone for 30 min before the addition of *E. coli* BioParticles. Treatment with P22 conjugated microparticles resulted in a 35% increase in phagocytosis. This increase was not observed in CD33-deficient cells. Co-treatment with the phagocytosis inhibitor, cytochalasin D, blocked phagocytosis in all treatment groups. Data were obtained in triplicate from n = 3 independent experiments and represented as mean ± SEM. *p < 0.05; two-way ANOVA.

Previous studies have suggested that the CD33 V-set domain is responsible for Aβ uptake (Griciuc et al., 2013, Malik et al., 2013), but it is not known which region of the domain is responsible for this activity. Having shown that P22 binds specifically to the sialic acid-binding site of this domain we now have a chemical tool to answer the question of whether the sialic acid-binding site is the appropriate target for small molecule ligands that might promote Aβ uptake. Siglecs preferentially recognize multivalent presentation of their ligands (Rillahan et al., 2014, Büll et al., 2016), and for this reason we conjugated the biotinylated form of P22 to microparticles labeled with both FITC and streptavidin (P22 microparticles) (see Transparent Methods). More than 60% CD33-transfected BV2 cells were positive for P22 microparticles compared with only 30% cells that were only incubated with control particles (unconjugated to P22) (Figure 4A). Although some P22 microparticles also bind to non-transfected cells, the difference remains significant for the cells transfected with CD33. This led us to believe that the compound bound preferentially to CD33 (Figure 4A). After showing that the P22 microparticles bind to CD33, we studied the effect of P22 on phagocytosis. For this, Aβ42 was labeled with pHrodo and added to CD33-transfected cells that had previously been incubated with P22 and control microparticles. The quantification of Aβ uptake shows that there is an increase of almost 20% in phagocytosis in transfected cells that have been treated with P22 microparticles with respect to those incubated without P22 (Figure 4B). As a control, we tested the core structural unit of P22, i.e., 3′-sialyllactose (Figure 2A), a much weaker binder of CD33 (millimolar affinity) and saw no difference in phagocytosis even when cells were pre-treated with millimolar concentration of the ligand. To further confirm our observations in murine BV2 cells, we examined the ability of P22 to modulate phagocytosis in the human THP-1 cell line in both wild-type and CD33 knockout cells. Differentiated THP-1 cells were either left untreated or were pre-treated with P22 monomers, P22 conjugated to microparticles, or microparticles alone for 30 min before the addition of *E. coli* BioParticles. Treatment with P22 monomers had no effect on the phagocytosis of the *E. coli* BioParticles; however, when P22 was conjugated to microparticles we observed a 35% increase in phagocytosis. Treatment with microparticles alone had no effect. The increase in phagocytosis with P22 conjugated microparticles was not observed in CD33-deficient THP-1 cells (Figure 4C). Co-treatment with the phagocytosis inhibitor, cytochalasin D, blocked phagocytosis in all treatment groups (Figure 4C). In summary, P22 increases phagocytosis in cells that express CD33.

### Concluding Remarks

Structural insights presented in this study reveal the molecular interactions that underpin sialic acid ligand recognition by CD33. We have shown that the sialic acid-based compound P22, when presented on microparticles, can increase phagocytosis of microglial cells and increase Aβ uptake into these cells. A link between CD33-like Siglecs (Siglec-9, Siglec-10, and Siglec-11) and their engagement of sialylated ligands resulting in inhibition of the innate immune response, including phagocytosis, is well established (Carlin et al., 2009, Chen et al., 2009, Wang and Neumann, 2010). The data suggest that these surface receptors can be neuroprotective by dampening immune response and neuronal damage in neuroinflammation. In contrast, the recent genetic data discussed above points to a protective effect of CD33 dysfunction in AD.

The sialic acid-binding site on CD33 is a promising target for developing therapeutics to promote clearance of the toxic Aβ peptide. The results presented here will be of great value in future structure-based drug discovery efforts targeting CD33 in AD. Inhibition of glycoprotein or glycolipid binding by CD33 may also be a useful therapeutic approach for other neurodegenerative diseases. For example, a Parkinson disease risk allele is correlated with increased expression of CD33 (Chan et al., 2016) and CD33 expression is differentially regulated in a murine model of amyotrophic lateral sclerosis (Ferraiuolo et al., 2007).

### Limitations of the Study

This study did not investigate the mechanism of action of P22 on CD33-mediated microglial uptake of Aβ ligands. We speculate that the mechanism of action of small molecule CD33 ligands might be to inhibit engagement of microglia with heavily sialylated ligands on neuritic plaques, thus releasing microglia to clear toxic Aβ species around the plaque. It remains unclear whether P22 microparticles act as an agonist or antagonist of CD33.

## Methods

All methods can be found in the accompanying Transparent Methods supplemental file.

## Supplemental Information

Document S1. Transparent Methods, Figures S1–S4, and Tables S1–S4
