# Supplementary material for: Small Molecule Binding to Alzheimer Risk Factor CD33 Promotes Aβ Phagocytosis
Source: iScience. 2019 Jul 19;19:110–8. doi: 10.1016/j.isci.2019.07.023 (PMC6669322; doi:10.1016/j.isci.2019.07.023)
Supplement: Document S1. Transparent Methods, Figures S1–S4, and Tables S1–S4 [file mmc1.pdf]

ISCI, Volume *19*

Supplemental Information

Small Molecule Binding to Alzheimer Risk Factor CD33 Promotes Aβ Phagocytosis

Luke A. Miles, Stefan J. Hermans, Gabriela A.N. Crespi, Jonathan H. Gooi, Larissa Doughty, Tracy L. Nero, Jasmina Markulić, Andreas Ebneth, Berthold Wroblowski, Daniel Oehlrich, Andrés A. Trabanco, Marie-Laure Rives, Ines Royaux, Nancy C. Hancock, and Michael W. Parker

SUPPLEMENTAL INFORMATION

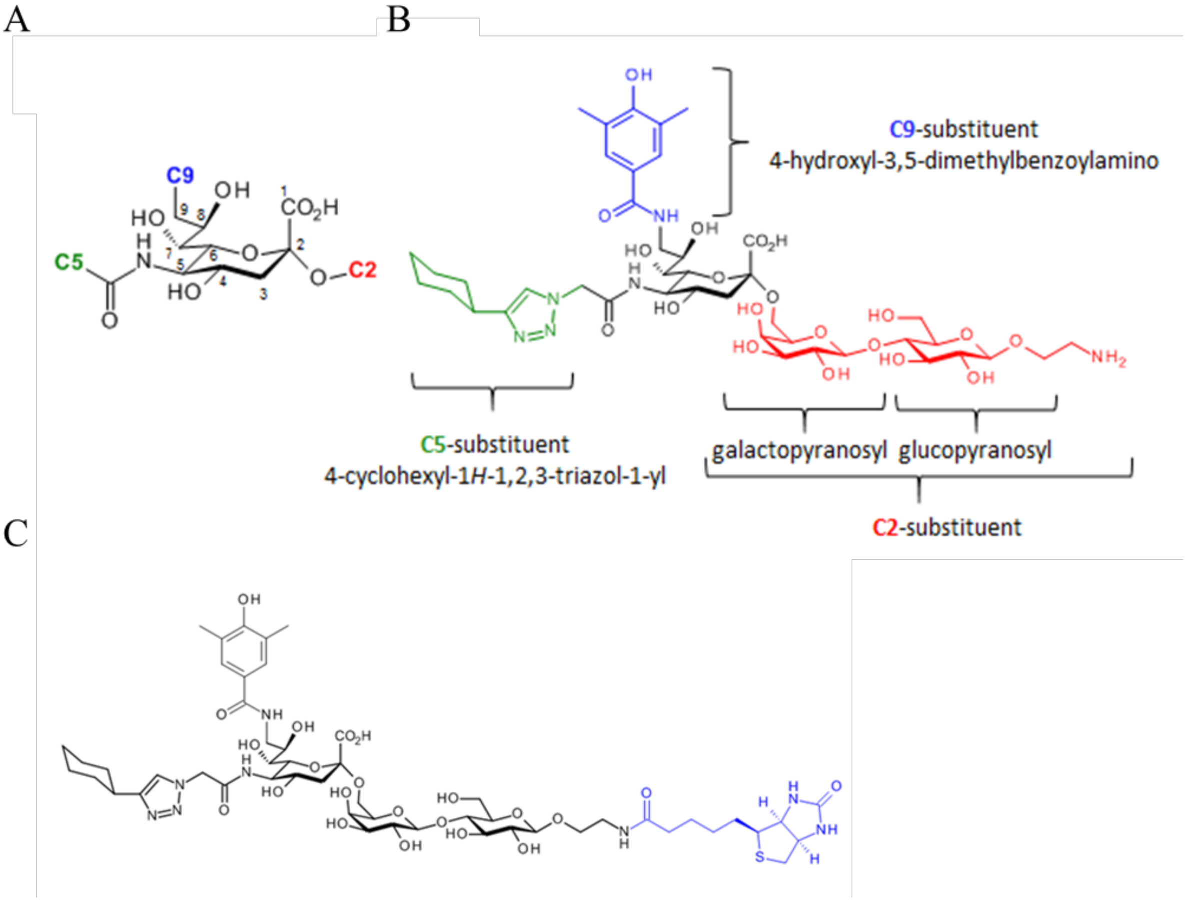

Figure S1. Chemical Structure of P22, related to Figure 2
(A) Numbering convention for the carbon backbone of the P22 sialic acid moiety.
(B) Chemical structure of P22 (β-D-glucopyranoside, 2-aminoethyl *O*-[*N*-[2-(4-cyclohexyl-1*H*-1,2,3-triazol-1-yl)acetyl]-9-deoxy-9-[(4-hydroxy-3,5-dimethylbenzoyl)amino]-α-neuraminosyl]-(2→6)-*O*-β-D-galactopyranosyl-(1→4)-; CAS registry number [1809735-27-0]).
(C) Biotinylated P22 (biotin label in blue).

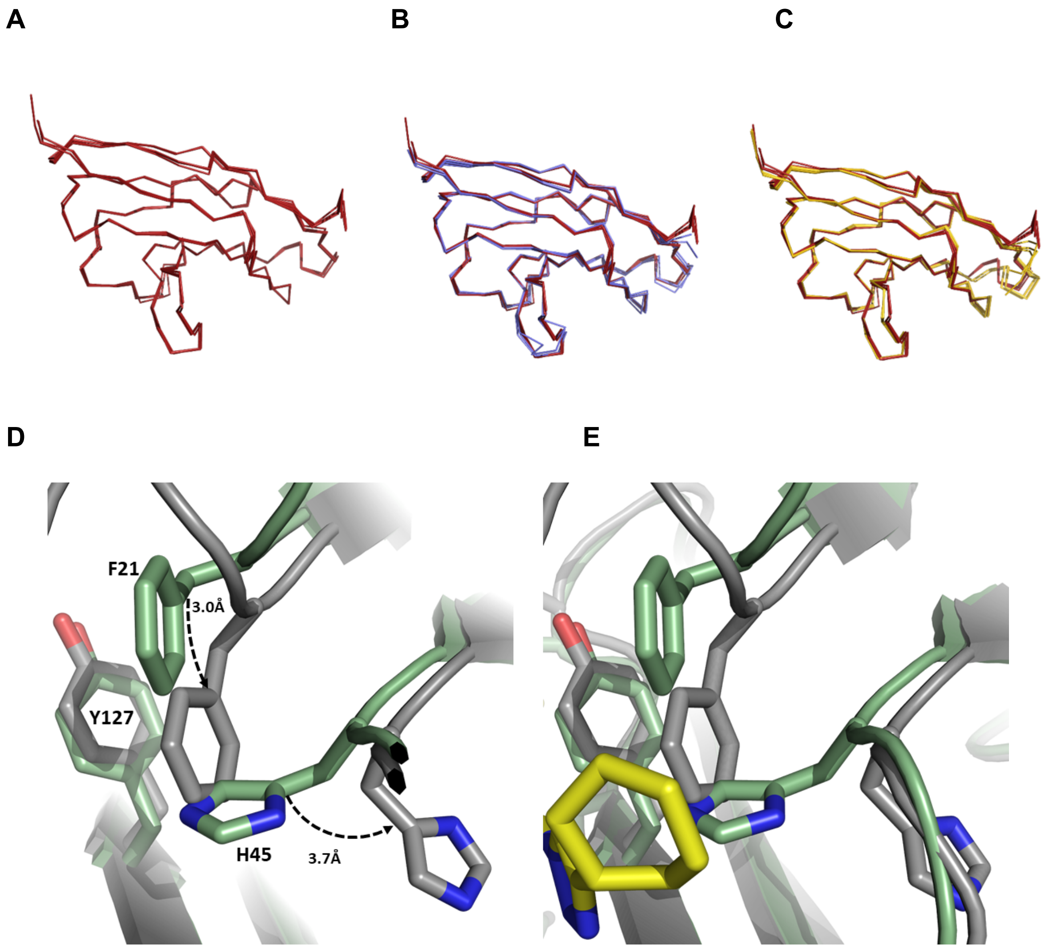

Figure S2. Comparison of Ligand Bound and Unbound CD33 V-set Domain Structures, related to Figure 2

(A) Alignment of the four CD33 chains identified in the unliganded structure, colored red. Maximum RMSD of matched Cα atoms between chains calculated to be 0.39-Å.

(B) Alignment of all four CD33 molecules in the unliganded structure, colored red, and available CD33 structures deposited in the PDB (PDB: 5IHB, 5J0B, 5J06), colored blue. Maximum rmsd of matched Cα residues calculated to be 0.41-Å to 5IHB chain B.

(C) Alignment of all four CD33 molecules in the unliganded structure, colored red, and all four P22 bound CD33 molecules identified across both co-crystal structures reported, colored yellow. Maximum rmsd of matched Cα residues between chains calculated to be 0.62-Å.

(D) Conformational changes on P22 binding to CD33. The unliganded CD33 structure is shown in green sticks and the P22 complex structure in grey sticks. Magnitude of displacement of aromatic side-chains is shown.

(E) Location of P22 (yellow sticks) in relation to the aromatic cluster.

A

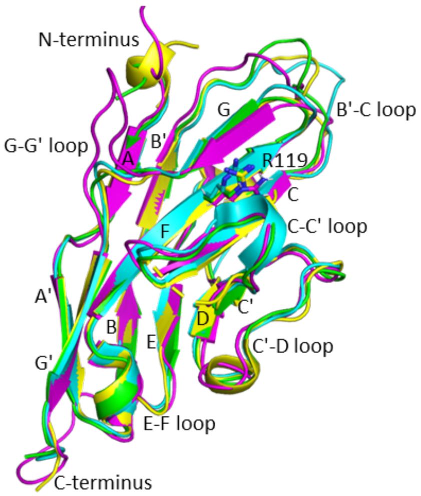

B

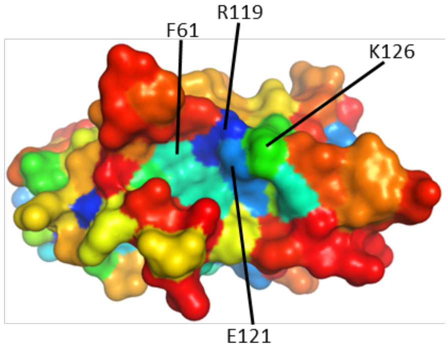

C

Human Siglec	1	2	4	5	6	7	8	9	10	11	12	14	15	16
% Sequence identity with CD33	22	25	28	56	60	55	58	58	44	41	53	57	26	41

D

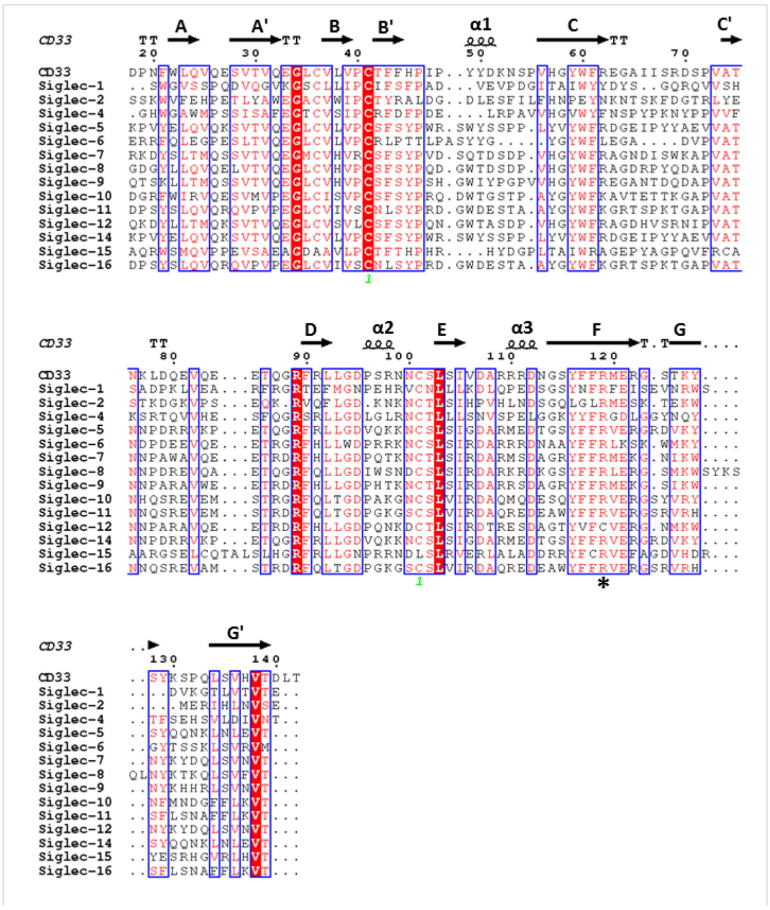

Figure S3. Comparison to Other Human Siglec V-set Domains, related to Figure 1
(A) Superimposition of the unliganded structure of CD33 (Siglec-3; green) with Siglec-5 (PDB: 2ZG2, cyan) (Zhuravleva et al., 2008), Siglec-7 (PDB: 1NKO, yellow) (Dimasi et al., 2004) and Siglec-8 (PDB:2N7A, magenta) (Pröpster et al., 2016). The lectin fold shows highly conserved β-strands labeled from the N-terminus forward as A, A', B, B', C, C', D, E, F, G and G'. The side-chains of the highly conserved R119 (numbering from CD33) are shown as sticks near the end of the central F-strand.

(B) The surface of CD33 with sequence variation between human Siglecs shown as a color spectrum from blue (most conserved) to red (least conserved), based on the alignment shown in panel D.
(C) Amino acid sequence identities between human Siglecs.
(D) Sequence alignment of human Siglecs using Clustalw (Thompson et al., 1994) and ESPript3.0 (Robert and Gouet, 2014). The β-strands and α-helices for CD33 are labeled above the sequence, the location of the C41-C101 disulfide bond is indicated by the green number 1 below the Siglec-16 sequence. The conserved arginine residue is indicated by the asterix. Sequence numbering is for CD33.

A

B

C

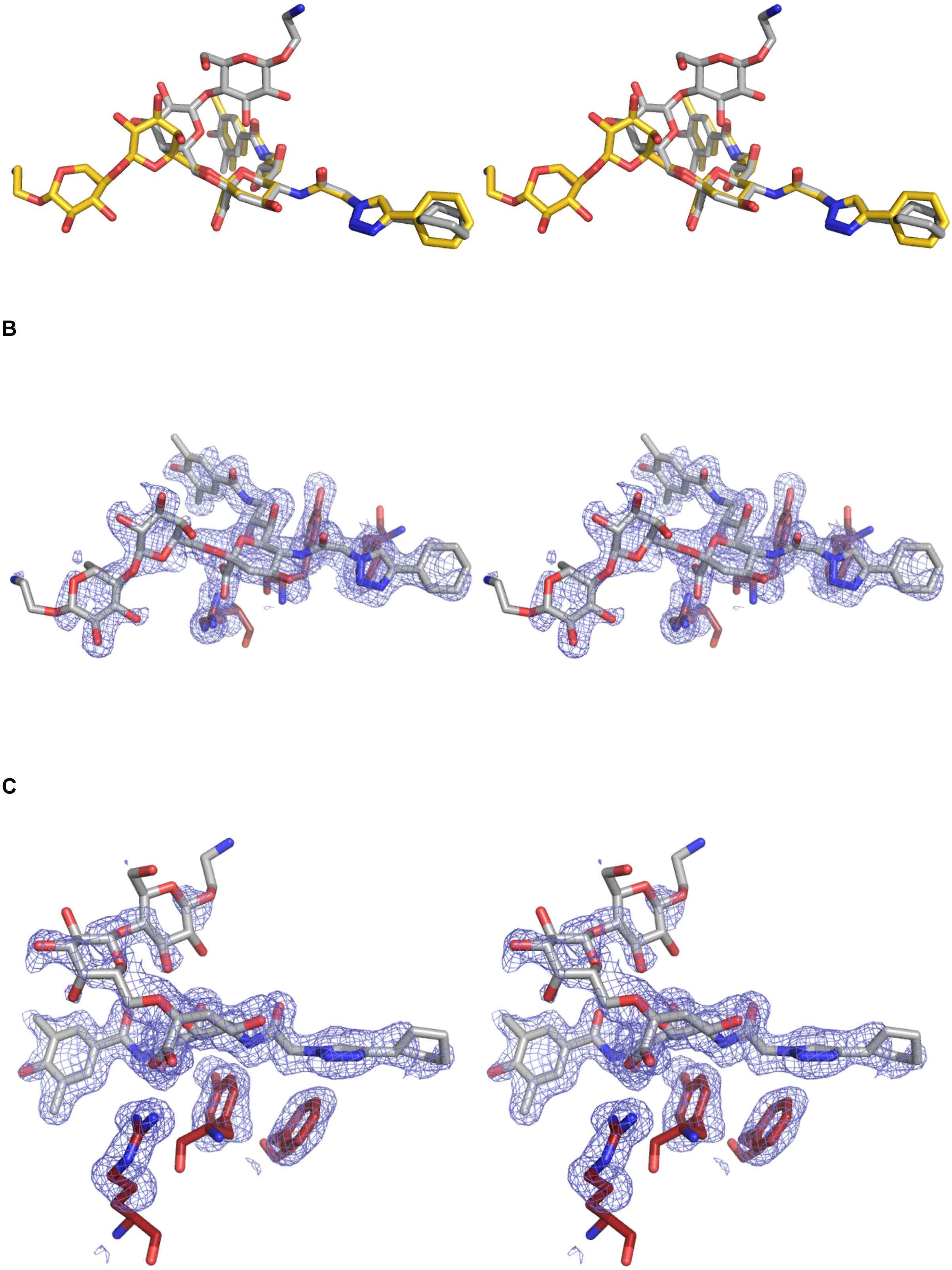

Figure S4. Structures of P22 Bound to CD33, related to Figure 2
(A) Stereo view of the two P22 binding modes (binding mode 1 in yellow and binding mode 2 in grey) observed in the crystal structures of P22 bound to CD33.
(B) Stereo simulating annealing omit map of binding mode 1 (seen in Crystal form no. 1) after the P22 molecules and the side-chains beyond the Cβ of Phe21, Tyr127 and Arg119 were removed before

refinement and calculation of the map. CD33 side-chains are shown in brown stick and the P22 molecule in grey stick, all with element coloring. The F_o-F_c map is shown in blue, contoured to 2.0 σ. (C) As for panel B but showing binding mode 2 (Crystal form no. 2).

SUPPLEMENTAL TABLES

Table S1. Crystallographic Data Processing and Refinement Statistics, related to Figures 1 and 2

Data Collection	P22 complex Crystal no. 1	P22 complex Crystal no. 2	Unliganded
Temperature (K)	100	100	100
Space group	$P2_1$	$P2_1$	$P2_1$
Unit Cell			
a, b, c (Å)	29.4, 68.8, 73.5	29.4, 68.3, 73.6	60.8, 41.5, 107.5
α, β, γ (°)	90, 97.0, 90	90, 97.5, 90	90, 96.6, 90
Resolution (Å)	36.46 - 1.80 (1.84 - 1.80)	34.17 - 1.75 (1.78 - 1.75)	42.48 - 1.78 (1.81 - 1.78)
No. of observations	141033 (8031)	110012 (5510)	171891 (8687)
No. of Unique Reflections	26760 (1534)	28662 (1477)	51251 (2673)
Redundancy	5.3 (5.2)	3.8 (3.7)	3.4 (3.2)
Data Completeness (%)	99.4 (94.8)	97.5 (88.9)	98.5 (90.3)
I/σ_I	12.6 (2.3)	13.4 (2.0)	14.7 (3.1)
R_{merge} (%)	7.3 (59.9)	6.1 (67.6)	4.4 (39.1)
Refinement Statistics			
Resolution (Å)	34.39 - 1.80	34.18 - 1.75	35.60 - 1.78
R_{work}	0.184	0.177	0.182
R_{free}	0.211	0.202	0.220
Non-Hydrogen Atoms			
Protein	2055	2023	4056
Water	253	333	490
r.m.s. deviations from ideality			
Bond angles (°)	1.154	1.001	0.998
Bond lengths (Å)	0.007	0.007	0.007
Average B-factor (Å^2)			
Protein	26.8	20.7	26.6
Water	38.1	33.0	35.8
Ramachandran Plot			
Residues in most Favored Regions (%)	98.4	100	98.2
Outliers (%)	0	0	0

Table S2. P22 Interactions with CD33[a], related to Figures 1 and 2

P22	CD33
**Sialic Acid**	
Carboxylate	Salt bridge to R119 guanidino moiety
**C5-Substituent**	
Amide	Hydrogen bond to backbone carbonyl of K126
Amide	vdW with Y127
Triazole ring	π-π edge-to-face interaction with F21
**C8-Substituent**	
Hydroxyl	Hydrogen bond to backbone amide of S128
**C9-Substituent**	
Amide	Hydrogen bond to backbone carbonyl of S128
4-hydroxy-3,5-dimethylbenzoylamino	vdWs with F117, K130, Y127, Y129

[a]Interactions only shown if they occur in all P22-CD33 structures.

Table S3. SPR Data for the Binding of CD33 and P22, related to Figure 3

Ligand	Analyte	k_{a1} (1/Ms)	k_{d1} (1/s)	K_{D1} (μM)	k_{a2} (1/RUs)	k_{d2}(1/s)	analysis model
$CD33^{WT}$	P22			118.0±41			1:1 steady state
$CD33^{C36S}$	P22			95.2±39			1:1 steady state
P22-biotin	$CD33^{WT}$	0.7±0.2E+05	1.4±0.4	18.0±01	3.2±2E-05	3.3±0.4E-02	Bivalent analyte
P22-biotin	$CD33^{C36S}$	0.4±0.2E+05	2.5±0.9	67.5±15			1:1 binding

Measurements in triplicate showing mean ± S.D.

Table S4 Effect of CD33 Mutations on P22-Alexa647 Binding Affinity, related to Figure 3

Variant	K_D (μM ± S.D.)	Fold-decrease compared to WT
WT	2.6 ± 1.1	n.a.
R119A	>100[****a]	>30
Y127A	>100[****a]	>30
S131A	3.1 ± 2.1[n.s.]	1.2
H45A	13.1 ± 10.3 [n.s.]	5
F21A	15.0 ± 4.5[*‡]	~6

n.a. not applicable
n.s. not significantly different compared to wild-type (WT)
[a]Significantly different from WT (* $p < 0.0332$; ** $p < 0.0021$; *** $p < 0.0002$; **** $p < 0.0001$)
K_D data are representative of n = 3 independent experiments performed in triplicate and presented as the average ± S.D. Statistical significance compared to WT was determined by one-way ANOVA with Dunnett post hoc analysis using GraphPad Prism 7.0 (GraphPad Software, Inc., La Jolla, CA, 92037, USA).

TRANSPARENT METHODS

Synthesis of P22

P22 (β-D-Glucopyranoside, 2-aminoethyl *O*-[*N*-[2-(4-cyclohexyl-1*H*-1,2,3-triazol-1-yl)acetyl]-9-deoxy-9-[(4-hydroxy-3,5-dimethylbenzoyl)amino]-α-neuraminosyl] (2→6)-*O*-β-D-galactopyranosyl-(1→4)-; CAS registry number [1809735-27-0]) was synthesized as described by Paulson and co-workers (Rillahan et al., 2014). Biotinylated P22 (Figure S1C) was prepared by treatment of a solution of P22 (12 mg, 0.012 mmol) in dimethylformamide (1 mL), with DIPEA (8.6 µL, 0.49 mmol) followed by addition of (+)-biotin N-hydroxysuccinimide ester (4.2 mg, 0.012 mmol), the resulting reaction mixture was then stirred for 2 h at RT. After which the reaction mixture was concentrated *in vacuo* and purified by reverse phase, performed via preparative HPLC (Stationary phase: RP XBridge Prep C18 OBD- 5μm,19x150mm, Mobile phase: 0.25% NH_4HCO_3 solution in water, CH_3CN) to afford Biotin-P22 (9 mg, yield 60.9%).

Protein production

Codon optimized (*E. coli*) DNA encoding D18 to H143 of human CD33 was synthesized and sub-cloned into a pET-30a+ vector with an N-terminal octa-histidine tag and a TEV protease cleavage site by Genscript. For SPR studies, a non-cleavable C-terminal AVI-tag was inserted into the clone and the free surface cysteine residue (C36) was mutated to serine by Genscript. The plasmid vector was transformed into *E. coli* (BL21*) and grown in LB broth under kanamycin selection at 37 °C in 2 L batches, in an orbital shaking incubator. Cultures were induced at mid-log phase with the addition of IPTG to 0.2 mM; bacteria were cultured for a further 5 h and harvested by centrifugation. Pelleted bacteria were suspended in lysis buffer (20 mM Tris pH 7.4, 150 mM NaCl, 5 mM EDTA, DNAse 1) and lysed by passage through an EmulsiFlex C-5 high-pressure homogenizer (Avestin). Lysates were subjected to centrifugation (18,000 g for 30 min) to pellet inclusion bodies and cell debris. Insoluble fractions were resuspended using a tissue grinder in wash buffer 1 (20 mM Tris pH 8.0, 150 mM NaCl, 1% Nonidet p 40 substitute, 5 mM EDTA) and pelleted again by centrifugation. This was repeated and insoluble fractions were washed twice with wash buffer 2 (20 mM Tris pH 8.0, 2 M NaCl, 1% Nonidet p 40 substitute, 5 mM EDTA) and twice with wash buffer 3 (20 mM Tris pH 8.0, 150 mM NaCl, 5 mM EDTA). Washed pellets were then resuspended in solubilization buffer (20 mL of 6 M urea, 50 mM Tris pH 8.5, 500 mM NaCl, 5 mM imidazole) and left rotating overnight at 4°C. The suspension was then diluted 100-fold into refold buffer (400 mM arginine, 100 mM Tris pH 8.5, 200 mM NaCl, 0.5 mM oxidizing glutathione, 5 mM reducing glutathione) and left stirring for at least 60 h at 4 °C. Refold solutions were concentrated, and buffer exchanged into storage buffer (20 mM Tris pH 8.5, 200 mM NaCl) with 10 mM imidazole using a tangential flow system (Sartorius) and loaded onto a Ni-NTA column. Protein was washed, and then eluted with an imidazole concentration gradient up to 500 mM. Pooled protein elution fractions were then dialyzed into storage buffer and incubated for 36 h at 4 °C with 0.1 mg hexa-histidine-tagged TEV protease added at 0 and 18 h. Tag-cleaved samples were passed over a Ni-NTA column (to capture TEV and cleaved tag) and then the eluted, cleaved protein further purified using a HiLoad 26/60 Superdex S200 size exclusion chromatography column equilibrated with 20 mM Tris pH 8.5, 200 mM NaCl. Pure samples of CD33 were concentrated to 3-7 mg/mL and stored in 25 µL aliquots at -80°C until required.

Surface plasmon resonance

All surface plasmon resonance (SPR) experiments were performed in PBS-P+ (KCl 0.2 g/L, KH_2PO_4 0.2 g/L, NaCl 8 g/L, Na_2HPO_4 1.15 g/L, Tween 20 0.05%) with a flow rate of 60 µL/min at 25°C using a Biacore T200. His-tagged CD33 wild-type was captured on a Series S NTA chip (GE) via Ni^{2+}/NTA chelation then covalently immobilized using amine coupling chemistry (GE Healthcare Laboratory Guidelines 29-0057-17 AB). Biotinylated AVI-tagged $CD33^{C36S}$ was immobilized on a SAHC 1500M sensor chip (Xantec) via streptavidin capture, to an average density of 6500 resonance units (RU). In the reverse conformation assay, P22-biotin was captured to an average density of 570 RU.

For affinity analysis of P22 against captured $CD33^{C36S}$, P22 was diluted using a threefold dilution series, each concentration tested twice, from 243 µM to 3 µM and analysis was performed in duplicate using single cycle kinetics. For kinetic analysis of $CD33^{C36S}$ and His-tagged CD33 wild-type against captured P22-biotin, CD33 was diluted using a threefold dilution series, each concentration tested three times, from 9 µM to 0.03 µM and duplicate multiple cycle kinetics was performed. All samples were double referenced against a blank surface and buffer only cycles. All data were analyzed using Biacore T200 Evaluation Software Version 2.0.

SNAP-tag assay

The plasmid encoding human CD33 wild-type, tagged at its N-terminus with a Snap-tag, was generated by sub-cloning human CD33 ORF (NM_001772.3), lacking its signal peptide, into a pT8-SNAP vector (CisBio # PT8SNAPZEO) using EcoRV and XhoI. The Snap-tagged CD33 mutants were generated by site-directed mutagenesis.

HEK293T cells were cultured in DMEM (ThermoFisher Scientific # 11965092) containing 10% FBS (Hyclone # SH30070.03) and 1% Penicillin-Streptomycin (ThermoFisher Scientific # 15140122) and transfected using Fugene HD (Promega # E2312) in 10 cm plates. For each plate, the DNA (µg):Fugene HD (µL) mix ratio was 1:3 and 5 million cells were added on top of 1 mL mix and incubated at 37°C in a CO_2 incubator for 48 h. Forty-eight hours after transfection, the Snap-tag was labeled by directly incubating the cells in 10 cm plates with 50 nM BG-K Terbium (CisBio # SSNPTBX) in complete media for 1 h at 37°C. After labeling, the cells were washed twice with 1X labeling media (CisBio # LABMED). The cells were then resuspended using Detachin (Amsbio # T100106). The cells were counted, centrifuged and resuspended in PBS at a concentration of 1,000 cells/µL and 10 µL were plated into each well. Increasing concentrations of P22-Alexa647 were incubated on top of the cells for 1 h at 4°C. Non-specific binding was assessed by competition with 100 µM of the non-labeled P22, as well as in control (Snap-tagged dopamine D2R receptor)-transfected cells.

Fluorescence was read with a PHERAstar plate reader using an excitation of 337 nm and emissions of 620 and 665 nm. Data presented are representative of three independent experiments performed in triplicates for each compound. Data are represented as the average ± S.D. Statistical significance was determined by one-way ANOVA with Dunnett post hoc analysis using GraphPad Prism 7.0 (GraphPad Software, Inc., La Jolla, CA, 92037, USA).

Aβ42 phagocytosis assay

A BV2 immortalized microglial cell line was cultured in DMEM low glucose with 10% (vol/vol) heat-inactivated FBS. Cells were seeded in a 12 well plate at 3×10^5 cells/mL and transfected using 0.9 µg human CD33 mRNA (hCD33) and 0.75 µL Lipofectamine® MessengerMAX (Invitrogen) per well.

Biotinylated P22 was dissolved in Hanks' Balanced Salt Solution (HBSS). 50 µg of P22 was added to 1 mg of FITC streptavidin particles (PC-SAFY-0.5, Kisker Biotech) that were previously equilibrated with HBSS buffer. After overnight incubation at 4°C, particles with and without compound were centrifuged (2,000 xg) and washed one time with HBSS. Microparticles were resuspended in 1 mL HBSS and were stored at 4 °C and protected from the light.

Aβ42 lyophilized stock was dissolved in 60 mM NaOH:ddH2O:10XPBS, in that order, in the ratio of 2:7:1. (The high pH buffer was used to prevent aggregation). 4 µL of dye (Invitrogen pHrodo™ Red Microscale Labelling Kit) was used to label 100 µL (1 mg/mL) of Aβ42. Excess dye was removed using 900 µL of resin (Bio-Gel p-6 fine resin suspended in PBS). Peptide was checked on an SDS-gel for the presence of aggregates.

Twenty four hours after transfection, cells were incubated with either 150 µL/mL FITC streptavidin particles alone or conjugated to biotinylated P22 in serum free media at 37°C in a CO_2 incubator for 30 min. After P22 incubation, 2 µg/mL of Aβ42 pHrodo was added for 1 h at 37°C in a CO_2 incubator. Cells were extensively washed with cold PBS and harvested using 200 µL PBS-EDTA for further analyses using flow cytometry. Ten thousand counts were acquired on an LRS Fortessa Flow Cytometer (Beckman). Samples were analyzed using FlowJo version 10. Data were collected in triplicate from n = 3 independent experiments and represented as the mean ± SEM. Statistical analysis was performed using GraphPad Prism 7 software. Comparisons involving more than two groups used one-way ANOVA followed by Tuckey post hoc test. All differences were considered significantly different when $p<0.05$.

Generation of CD33 knockout THP-1 cells

Guide RNA sequences for CRISPR/Cas9 were designed online using the Broad Institute sgRNA design tool at https://portals.broadinstitute.org/gpp/public/analysis-tools/sgrna-design. The gRNA selected for the KO of CD33 targets exon 3 of the human CD33 gene (target sequence: 5'-GGCCGGGTTCTAGAGTGCCA *GGG*-3'; PAM in italics). crRNA and tracrRNA oligos as well as recombinant Cas9 protein were ordered from Integrated DNA Technologies (IDT). The Cas9RNP complexes were made by assembling the 3 components, crRNA:tracrRNA:Cas9, at a 1.25:1.25:1 ratio. Cas9RNP were electroporated at a final concentration of 3.3 µM into 300,000 THP-1 cells using the Neon electroporation device (ThermoFisher Scientific) programmed at 1600 volts, 10 milliseconds and 3 pulses. Eight days after electroporation, cells were stained with PE conjugated mouse anti-human CD33 (Pharmigen cat #555450) and the CD33 negative population was collected by fluorescence-activated cell sorting (FACS) with a FACSCanto™ II cell sorter (BD BioSciences).

THP-1 phagocytosis assay

THP-1 cells (control and CD33KO) were differentiated in growth media (RPMI 1640, 10% FCS) supplemented with 100 ng/mL PMA for 3 days. Following this, cells were returned to normal growth media for an additional 3 days. Differentiated cells were then lifted, counted and 75,000 cells/well were plated in a 96 well plate. Cells were left for 1 h to settle then transferred to serum free media for 2 h. Following this, cells were incubated with P22 monomer, P22 conjugated microparticles or microparticles alone for 30 min prior to incubation with pHrodo *E. coli* Bioparticles for 2 hours per the manufacturer's instruction. All treatments were present for the duration of the experiment. Mean fluorescent intensity (MFI) was determined using a Perkin Elmer Enspire Plate Reader.

Crystallization

Thawed CD33 at 3.6 mg/mL was supplemented with 1 mM P22 and screened against the MCSG1 Suite (Microlytic) in 200 x 200 nL sitting drops, dispensed by a Gryphon LCP (Art Robbins Instruments) crystallization robot. CD33 crystals grew with a well solution of 30% w/v PEG MME 550, 100 mM HEPES pH 7.5, 50 mM $MgCl_2$ at 22°C (Crystal form no.1) and presented in a range of cubic morphologies from 40 – 80 µm in length. Initial hits were reproduced in 2 x 2 µL hanging drops appearing over a week. Similar shaped crystals also grew from a well solution of 18% w/v PEG 4000, 100 mM MES pH 6.5, 600 mM NaCl at 22°C (Crystal form no. 2). Crystals were briefly transferred to a cryo-solution of the mother liquor supplemented with 20% (v/v) glycerol before being flash frozen directly into liquid nitrogen.

Unliganded CD33 crystals were likewise grown from a protein batch concentrated to 15 mg/mL and screened against The PEGs Suite (Qiagen). Initial hits were reproduced in 2 x 2 µL hanging drops appearing overnight. These crystals grew using well solution composed of 25% w/v PEG 4000, 100 mM Tris-HCl pH 8.5 at 22°C but were small and excessive nucleation was apparent despite being well-shaped. The best crystals were achieved with the same well solution made up with 10% (v/v) glycerol; cubic-shaped crystals appeared overnight or soon after and increased in size over a week or so. Crystals were flash frozen directly from the mother liquor.

Structure determination

X-ray diffraction data were acquired on the MX beamlines at the Australian Synchrotron (Clayton, Victoria, Australia) (Cowieson et al., 2015; Aragão et al., 2018) using Blue-Ice software (McPhillips et al., 2002). Data sets were acquired at a single wavelength of 0.9537-Å, in a nitrogen cryostream (100 K). Diffraction images were integrated using XDS (Kabsch, 2010) and scaled with Aimless of the CCP4 program suite (Winn et al., 2011), setting aside 5% of the reflections for the R_{free} set. Data collection statistics are shown in Table S1.

The initial unliganded structure was determined by molecular replacement with Phaser from the Phenix software suite (Adams et al., 2010) in the $P2_1$ space group. A successful molecular replacement solution was achieved with a probe model derived from the Siglec-7 structure (PDB:1NKO (Dimasi et al., 2004)), identifying four CD33 molecules in the asymmetric unit. All other CD33 structures were determined by molecular replacement with Phaser in the $P2_1$ space group using the unliganded structure as the search model. Refinement and rebuilding were performed with Coot (Emsley and Cowtan, 2004) and Phenix Refine (Adams et al., 2010). Structure validation was monitored with MolProbity (Chen et al., 2010). The quality of the final electron density maps for the P22 complex structures is shown in Figure S4. Refinement statistics are shown in Table S1. The final models have better than 98.2% of residues in favored regions of the Ramachandran plot with no outliers. Structural figures were produced using PyMOL version 1.8.2.2 (http://www.pymol.org), chemical structures were drawn using MarvinSketch 6.0.2 (http://www.chemaxon.com), and interactions between CD33 and P22 were identified and visualized using LigPlus (Laskowski and Swindells, 2011) version 1.3.5 (www.ebi.ac.uk/thornton-srv/software/LigPlus).

QUANTIFICATION AND STATISTICAL ANALYSIS

All results are presented as either average ± S.D. or mean ± SEM. Microsoft Excel and GraphPad Prism 7 software were used for statistical analysis. Data were analyzed using one-way ANOVA, or repeated-measures one-way ANOVA, followed by Tukey's or Dunnett's multiple comparisons test, as specified in the Figure legends. Only two-sided tests were used, and all data analyzed met the assumption for the specific statistical test that was performed. Probability levels of $p < 0.05$ were considered statistically significant.

DATA AND SOFTWARE AVAILABILITY

Atomic coordinates and structure factors of the unliganded CD33 V-set domain and the two P22 complex models have been deposited in the Protein Data Bank under the accession numbers 6D48, 6D49 and 6D4A, respectively.

EXPERIMENTAL MODEL AND SUBJECT DETAILS

Cell Lines

Chemically competent *E. coli* BL21cells used to produce recombinant hCD33 proteins were obtained from Thermo Fisher Scientific. BV2 cells used for the phagocytosis assay were a gift from Prof Kevin Barnham, Florey Institute of Neuroscience and Mental Health (Parkville, Australia). HEK293T cells used for the SNAP-tag assay were obtained from and authenticated by ATCC. THP-1 cells used for the phagocytosis assay were obtained from and authenticated by ATCC and by Janssen using STR analysis. Sex information of HEK293T cell lines is not available. THP-1 cell lines are derived from a male donor.

CONTACT FOR REAGENT AND RESOURCE SHARING

Further information and requests for resources and reagents should be directed to and will be fulfilled by the Lead Contact, Michael Parker (mparker@svi.edu.au). All modified cell lines and plasmids of mutants will require completion of a materials transfer agreement with Janssen Pharmaceuticals. There are no fresh supplies available of the tool compound, P22, but the interested reader is directed to the corresponding author of the original paper describing its synthesis (Rillahan et al., 2014).

Supplemental References

Adams, P.D., Afonine, P.V., Bunkóczi, G., Chen, V.B., Davis, I.W., Echols, N., Headd, J.J., Hung, L.W., Kapral, G.J., Grosse-Kunstleve, R.W., et al. (2010) PHENIX: a comprehensive Python-based system for macromolecular structure solution. Acta Crystallogr. D Biol. Crystallogr. *66*, 213-221.

Aragão, D., Aishima, J., Cherukuvada, H., Clarken, R., Clift, M., Cowieson, N.P., Ericsson, D.E., Gee, C.L., Macedo, S., Mudie, N. et al. (2018) MX2: a high-flux undulator microfocus beamline serving both the chemical and macromolecular crystallography communities at the Australian Synchrotron". J. Synchrotron Rad. *25*, 885-891.

Chen, V.B., Arendall, W.B. 3rd, Headd, J.J., Keedy, D.A., Immormino, R.M., Kapral, G.J., Murray, L.W., Richardson, J.S., and Richardson, D.C. (2010) MolProbity: all-atom structure validation for macromolecular crystallography. Acta Crystallogr. D Biol. Crystallogr. *66*, 12-21.

Cowieson, N.P., Aragão, D., Clift, M., Ericsson, D.J., Gee, C., Harrop,S.J., Mudie, N., Panjikar, S., Price, J.R., Riboldi-Tunnicliffe, A. et al. (2015) MX1: a bending-magnet crystallography beamline serving both chemical and macromolecular crystallography communities at the Australian Synchrotron. J. Synchrotron Rad. *22*, 187-190.

Emsley, P., and Cowtan, K. (2004) Coot: model-building tools for molecular graphics. Acta Crystallogr. D Biol. Crystallogr. *60*, 2126-2132.

Kabsch, W. (2010) XDS. Acta Crystallogr. D Biol. Crystallogr. *66*, 125-132.

Laskowski, R.A., and Swindells, M. (2011) LigPlot+: multiple ligand-protein interaction diagrams for drug discovery. J. Chem. Inf. Model. *51*, 2778-2786.

McPhillips, T.M.., McPhillips, E., Chiu, H.J., Cohen, A.E., Deacon, A.M., Ellis, P.J., Garman, E., Gonzalez, A., Sauter, N.K., Phizackerley, R.P., et al. (2002) Blu-Ice and the Distributed Control System: software for data acquisition and instrument control at macromolecular crystallography beamlines. J. Synchrotron Radiat. *9*, 401-406.

Robert, X., and Gouet P. (2014) Deciphering key features in protein structures with the new ENDscript server. Nucleic Acids Res. *42*, W320-W324.

Thompson, J.D., Higgins, D.G., and Gibson, T.J. (1994) CLUSTAL W: improving the sensitivity of progressive multiple sequence alignment through sequence weighting, position-specific gap penalties and weight matrix choice. Nucleic Acids Res. *22*, 4673-4680.

Winn, M.D., Ballard, C.C., Cowtan, K.D., Dodson, E.J., Emsley, P., Evans, P.R., Keegan, R.M., Krissinel, E.B., Leslie, A.G., McCoy, A., et al. (2011) Overview of the CCP4 suite and current developments. Acta Crystallogr. D Biol. Crystallogr. *67*, 235-242.
